# Oral Treatment With an Engineered Uricase, ALLN-346, Reduces Hyperuricemia, and Uricosuria in Urate Oxidase-Deficient Mice

**DOI:** 10.3389/fmed.2020.569215

**Published:** 2020-11-24

**Authors:** Kateryna Pierzynowska, Aditi Deshpande, Nadiia Mosiichuk, Robert Terkeltaub, Paulina Szczurek, Eduardo Salido, Stefan Pierzynowski, Danica Grujic

**Affiliations:** ^1^Department of Animal Physiology, Kielanowski Institute of Animal Nutrition and Physiology Polish Academy of Sciences, Jabłonna, Poland; ^2^Department of Biology, Lund University, Lund, Sweden; ^3^SGP+Group, Trelleborg, Sweden; ^4^Allena Pharmaceuticals, Newton, MA, United States; ^5^Department of Biochemistry and Biotechnology, Vasyl Stefanyk Precarpathian National University, Ivano-Frankivsk, Ukraine; ^6^VA Medical Center, University of California, San Diego, La Jolla, CA, United States; ^7^Department of Animal Nutrition and Feed Sciences, National Research Institute of Animal Production, Balice, Poland; ^8^Hospital Universitario de Canarias, Universidad La Laguna & Center for Rare Diseases (CIBERER), Tenerife, Spain; ^9^Department of Biology, Institute Rural Medicine, Lublin, Poland

**Keywords:** gout, CKD, urolithiasis, urate-lowering therapy, ABCG2

## Abstract

Limitations in efficacy and/or tolerance of currently available urate-lowering therapies (ULTs), such as oral xanthine oxidase inhibitors, uricosurics, and intravenous uricase agents contribute to the development of refractory gout. Renal excretion is the major route of uric acid elimination, but the intestinal tract plays an increasingly recognized role in urate homeostasis, particularly in chronic kidney disease (CKD) in which the renal elimination of urate is impaired. We targeted intestinal degradation of urate *in vivo* with ALLN-346, an orally administered, engineered urate oxidase, optimized for proteolytic stability, and activity in the gut. We tested ALLN-346 in uricase/urate oxidase deficient mice (URKO mice) with severe hyperuricemia, hyperuricosuria, and uric acid crystalline obstructive nephropathy. A total of 55 male and female URKO mice were used in the two consecutive studies. These seminal, proof-of-concept studies aimed to explore both short- (7-day) and long-term (19-day) effects of ALLN-346 on the reduction of plasma and urine urate. In both the 7- and 19-day studies, ALLN-346 oral therapy resulted in the normalization of urine uric acid excretion and a significant reduction of hyperuricemia by 44 and 28% when therapy was given with food over 24 h or was limited for up to 6 h, respectively. Fractional excretion of uric acid (FEUA) was normalized with ALLN-346 therapy. Oral enzyme therapy with engineered urate oxidase (ALLN-346) designed to degrade urate in the intestinal tract has the potential to reduce hyperuricemia and the renal burden of filtered urate in patients with hyperuricemia and gout with and without CKD.

## Introduction

Hyperuricemia, defined as a serum urate (sUA) concentration greater than either 6.8 or 7.0 mg/dl (1), affects around 20% of adults within the United States (2) and promotes the development of gout, which in the United States has a prevalence of ~4%. In gout, tissue deposits of monosodium urate crystals promote acute and chronic inflammatory arthritis (3). In addition, urolithiasis is common in gout and is promoted by decreased solubility of uric acid in the acidic pH of the urine and increased urinary uric acid concentration (4). Gout is associated with a decreased health-related quality of life and increased health care utilization (5). In addition, hyperuricemia has been identified as a potential contributor to the pathophysiology of multiple comorbidities associated not only with gout, but also with asymptomatic hyperuricemia. These include hypertension, chronic kidney disease (CKD), obesity, metabolic syndrome, type 2 diabetes, and coronary artery disease (6–9).

Although some gout patients have heritable conditions that cause the overproduction of uric acid (10), insufficient renal excretion of uric acid is by far the most frequent cause of hyperuricemia in gout (11). In healthy subjects, approximately two thirds of uric acid excretion is renal (12, 13). Urate is also secreted into the small intestine, where it can be degraded by gut bacteria (intestinal uricolysis) or eliminated by defecation (12, 14). In individuals with normal uric acid metabolism, approximately one third (~200 mg/day) of daily urate elimination occurs by intestinal secretion (12, 13) with the ability to increase urate elimination to ~300 mg/day.

In those with CKD, the fraction of urate eliminated intestinally may increase to between 50 and 70%, surpassing renal elimination (12, 15, 16). The importance of intestinal elimination of urate in patients with CKD has been supported by the observation that sevelamer, a non-absorbed phosphate binder, which is also a non-selective urate binder, significantly reduces sUA concentration in hyperuricemic patients on hemodialysis (17, 18). However, the magnitude of the sevelamer urate-lowering effects was only moderate, achieving a mean sUA reduction of ~0.6–0.7 mg/dl in a randomized clinical trial (17) with the highest sUA reduction observed in subjects with the most severe hyperuricemia.

Urate transport and the maintenance of urate homeostasis involves a complex network of transporters located in the kidneys and the intestine (19). Major transporters involved in renal urate disposition that are encoded by gout-risk genes include SLC22A12 (encoding URAT1), *SLC2A9* (encoding Glut9), and the urate secretory transporter *ABCG2* (ATP-binding cassette) (13). In contrast to the majority of renal urate transporters, *ABCG2* is also expressed in the small intestine (20), where it plays an important role in lowering sUA (21). Moreover, the specific impact of intestinal *ABCG2* on urate homeostasis appears to increase as renal function declines (15, 16). This is also illustrated in animal models in which normal sUA levels were maintained with increased intestinal *ABCG2* expression in healthy rats subjected to subtotal nephrectomy (22). Similarly, decreased intestinal urate elimination and hyperuricemia development (23) resulted from the knockout of the *ABCG2* transporter (*Abcg2* null mice). In humans, certain dysfunctional *ABCG2* variants are associated with hyperuricemia and increased risk of gout, including the early onset of gout disease and expression of the severe tophaceous gout phenotype (24). Such variants include the widely prevalent *ABCG2* Q141K (rs2231142), which has a particularly high allele frequency (up to ~30%) in multiple East Asian populations at large and, not unexpectedly, an even higher allele frequency (up to ~50%) in gout patients from those populations (25). Severe impairment of ABCG2 function causes hyperuricemia and gout associated with excessive renal urate filtration (“renal uric acid overload”) (24).

Hyperuricemia and gout are prevalent in patients with CKD relative to those with preserved renal function with the estimated prevalence of gout in patients with stage 3 CKD in the United States being between ~20 and 30% (2). This poses a unique challenge to clinicians seeking effective treatment for patients with CKD as both renal impairment and urolithiasis limits the use of existing urate-lowering therapies (ULTs), including uricosurics that increase uric acid excretion and also xanthine oxidase inhibitors due to increased risk of potential toxicity with the optimal therapeutic dose (26, 27). Taking into account the limitations of present therapies and the robust secretion of urate into the small intestine (12, 14), a new uricase, ALLN-346, was designed as a potentially safer alternative therapy that is not absorbed into circulation and has the potential to degrade the urate secreted and/or formed in the small intestine, thereby reducing the systemic urate burden. ALLN-346 was designed to achieve this outcome through the protein engineering of *Candida utilis* uricase to impart proteolytic stability while not decreasing the specific activity compared to the wild-type enzyme using proprietary ProteinGPS technology (Atum, CA, USA). Through a machine learning–guided molecular evolution process, the proteolytic stability of the uricase in the intestinal tract was enhanced, enabling it to degrade urate (U.S. Patent Application #20200071681).

The gene encoding uricase/urate oxidase (UrOx) was inactivated during evolution in humans, and to assess the efficacy of ALLN-346 in a “human-like” model of hyperuricemia, the UrOx knockout mouse model was used. The B6;129S7-Uoxtm1Bay/J mouse strain (URKO mice) (28) is characterized by severe hyperuricemia with an average plasma UA of 11.0 ± 1.7 mg/dl, which is ~12 times higher than that in the wild-type controls (0.9 ± 0.3 mg/dl) with active uricase liver enzymes. These mice have pronounced hyperuricosuria, and they spontaneously develop uric acid crystalline obstructive nephropathy (28). Our work demonstrates that oral treatment with ALLN-346 reduces both hyperuricemia and hyperuricosuria in URKO mice via effective degradation of urate secreted in the intestinal tract.

## Materials and Methods

Experiments were performed in accordance with the recommendations in the Guide for the Care and Use of Laboratory Animals of the National Institutes of Health. All experimental procedures were approved by the University of Lund Ethics Review Committee on Animal Experiments (Approval No. M14331-17). All efforts were made to minimize animal suffering during experimental procedures.

### Molecular Evolution of *Candida utilis* Uricase

Uricase mutants were developed using protein-engineering principles by a synthetic biology company ATUM (CA, USA). Briefly, DNA fragments encoding the 95 mutant *C. utilis* uricases were cloned into a rhamanose pD861-NH expression vector with an N-terminal His-tag. Following expression in *Escherichia coli* cells, recombinant mutants were purified using an Ni-NTA column, according to standard procedures and further tested for enzymatic activity and stability in the presence of pancreatin (Sigma-Aldrich, Cat No. P7545). Briefly, 25 ng/μl of uricase was incubated with 20 ng/μl of pancreatin at 37°C for up to 200 min in simulated intestinal fluid buffer (50 mM potassium phosphate, pH 6.8) in a 96-well plate. Following incubation with pancreatin (Cat No. P7545, Sigma-Aldrich, St. Louis, MO, USA) for the indicated time points, the best mutants were selected based on the enzymatic activity using an absorption-based assay (enzymatic oxidation of uric acid to 5-hydroxyisourate by uricase results in a corresponding drop in 293 nm absorbance over time). The DNA and amino sequence of ALLN-346 chosen for further development is shown in the U.S. recombinant uricase enzyme patent application #20200071681.

The novel engineered uricase is the active compound of ALLN-346 with an optimal pH activity range of between 6.5 and 9; the enzyme is acid sensitive and is irreversibly inactivated below a pH of 5. Hence, in order to protect the uricase enzyme from the acidic pH of the stomach, ALLN-346 was administered as a food/enzyme mix together with 1% bicarbonate (w/w) as a basifier.

### Animals

A total of 55 male and female mice (strain: B6;129S7-Uoxtm1Bay/J) generated at Jackson Laboratory (USA) (https://www.jax.org/strain/002223) were used in the study. When the study was initiated, the mice were >6 weeks of age with a body weight of ~22–26 g. Mice were maintained on a 12-h day/night cycle with lights on from 7:30 a.m.−7:30 p.m. (07:30–19:30 h) and dark from 7:30 p.m.−7:30 a.m. (19:30–07:30 h), respectively. Prior to the experimental period, the mice were housed in groups in regular cages equipped with water bottles and feeders (6 mice/cage) with beta chip bedding. Allopurinol, which was needed to ensure mouse survival, was added to their drinking water at a dose of 150 mg/L (28). Following randomization into groups, the mice were individually housed in collection cages, equipped with a small feeding bowl and a drinking bottle. To label the mice, each cage was clearly identified with a color-coded card indicating the study number, animal number, sex, and group allocation. A standard certified commercial diet (standard rodent pelleted feed; Lactamin, Vadstena, Sweden) was provided *ad libitum* during the entire study period, including the adaptation period, during which mice were acclimatized to the collection cages. Drinking water was provided *ad libitum* as well. Animal feed intake during treatment was measured daily, and their body weight and water intake were measured weekly.

### Experimental Design and ALLN-346 Dosing

The experimental design comprised two consecutive studies, a short- (7-day) and a long-term (19-day) study. Both studies had a 7-day-long pretreatment period when allopurinol was withdrawn from the drinking water to induce hyperuricemia and hyperuricosuria. During this time, mice were also adapted to collection cages and to the special feed containing ALLN-346. In both studies, mice were randomized into groups based on plasma urate concentrations, measured from a single blood sample collected at the end of the pretreatment period irrespective of gender. The targeted daily dose of ALLN-346 was ~2,000 units/day.

In the initial 7-day study, 31 female and male mice with an average body weight of 26.3 ± 0.69 g were divided into four groups: control (*n* = 7, 3 male, 4 female), in which no treatment was given; ALLN-346 (*n* = 7, 5 male, 2 female), in which mice were given ALLN-346 mixed with food that was enriched with 1% sodium bicarbonate (basifier); and two allopurinol groups, ALLO 50 (*n* = 8, 3 male, 5 female) and ALLO 150 (*n* = 9, 4 male, 5 female), in which mice were treated with allopurinol at doses of 50 and 150 mg/l provided in their drinking water (28), respectively, and were also used as a comparator to the ALLN-346 group. The daily dose of ALLN-346 was calculated based on average daily food consumption. The ALLN-346 was administered in feeders during the 24-h cycle in the form of a “cake” prepared from regular chow powder rodent diet that was enriched with peanut butter and olive oil to provide a uniform food/enzyme mix. The food cake mix contained 100 g chow powder rodent diet, 50 g peanut butter, and ~12–25 g olive oil and was provided *ad libitum*. Adaptation to this diet without ALLN-346 addition occurred during the pretreatment period.

In the second, 19-day study, 24 female and male mice with an average body weight of 22.0 ± 2.89 g were divided into three groups: control (*n* = 7, 4 male, 3 female), no treatment; ALLO 90 (*n* = 7, 5 male, 2 female), in which mice were treated with allopurinol (90 mg/l in drinking water); and ALLN-346 (*n* = 10, 7 male and 3 female), in which mice were treated with ALLN-346 mixed with food enriched with 1% sodium bicarbonate as a basifier. ALLN-346 was offered as a “single feed” during 4–6 h at night in the form of a “milk-fat cake” consisting of a mixture of ~130 g of dry milk, ~30 g butter, 75 g cornstarch, 75 g corn powder, and ~80 g water mixed. The mice were adapted to this diet during the pretreatment period.

### Blood Collection

Blood samples (~50 μl) were collected from the submandibular vein into Microvette®100 LH tubes (SARSTEDT AG&Co. KG, Numbrecht, Germany). In general, blood samples were collected at the end of the pretreatment, treatment, and follow-up periods. After collection, blood samples were centrifuged at 3,000 g at 4°C for 10 min and analyzed fresh or stored for up to 48 h at 4°C until analysis.

### Urine Collection

A 24 h urine sample was collected into collection tubes 3 times during the pretreatment and 3 times during the treatment period. To prevent precipitation of salts of uric acid, 1–2 drops of NaOH (8 M) were added to the collection tubes. Urine samples were analyzed fresh or stored for up to 48 h at 4°C until analysis.

### Urate and Creatinine Measurement in Plasma, Urine, and Chyme

The concentration of urate in plasma and urine samples was measured spectrophotometrically, using an enzymatic Uric Acid Assay Kit Liquick Cor-UA 60 PLUS (cat. # 2-258, P.Z. Cormay S.A., Lomianki, Poland), according to the manufacturer's protocol. Before measurements, urine samples were diluted 5–15 times with distilled water, depending on the experimental group; the plasma samples were analyzed without dilution.

Urate analysis was also performed in chyme samples collected from the stomach, small intestine (duodenum, jejunum, and ileum), and colon of the mice at the end of the 19-day study. Samples were extracted with 0.068 M Li2CO3 (pH = 11.5) (1:3, weight: volume) and, following gentle mixing, were incubated in boiling water for 10 min, followed by 15 min centrifugation at 13,000 g at 4°C. The resultant supernatant was then filtered through the Centrifugal Concentrator Vivaspin 500, 10 kDa (Vivaspin®, Sartorius, Göttingen, Germany) at 15 min at 13,000 g and 4°C. The filtrate, with or without dilution, was used to determine urate concentrations, using the same kit as that used for urine and plasma urate determinations.

The concentration of creatinine in the urine and plasma was measured spectrophotometrically using a colorimetric creatinine assay kit (ab204537, Abcam, Cambridge, UK) and an enzymatic Creatinine Assay Kit Liquick Cor-CREA ENZYMATIC 60 (cat. # 2-267, P.Z. Cormay S.A., Lomianki, Poland), respectively, in accordance with the manufacturer's instructions.

### Calculation of Renal Test Parameters

Fractional excretion of urate (FEUA) as a ratio of creatinine and urate clearance, was calculated as follows:

$$\begin{array}{l} FEUA\;\left(\%\right)\\ =\frac{Urinary\;uric\;acid\;\left(\frac{mg}{dl}\right)\;\times \;Serum\;creatinine\;\left(\frac{mg}{dl}\right)}{Serum\;uric\;acid\;\left(\frac{mg}{dl}\right)\times \;Urinary\;creatinine\;\left(\frac{mg}{dl}\right)}\times \;100 \end{array}$$

### Statistical Analysis

Statistical analysis was performed using a repeated-measures ANOVA with the Geisser-Greenhouse correction followed by a Bonferroni correction for multiple comparisons. All analyses were carried out using Prism, version 8 (GraphPad Software, Inc., San Diego, CA, USA). Data are expressed as mean ± standard error of the mean (SEM). The distribution of the parameters was checked using a Shapiro-Wilk normality test. In all statistical analyses, *p* <0.05 was considered significant.

## Results

### Engineering Urate Oxidase Design and Selection

An analysis of pancreatin stability of 196 recombinant mutant *C. utilis* uricases generated during two rounds of molecular evolution was used to identify key substitutions that contribute to improved pancreatin stability. Results for *C. utilis* uricase mutants with the most improved pancreatin stability were confirmed over multiple protein preparations. Engineered UrOx was identified as the enzyme with 20-fold increased stability against pancreatin compared to the wild-type *C. utilis* uricase and without significantly decreased specific activity. Engineered UrOx exhibited a half-life of 85.3 min compared to 4.3 min for the wild-type *C. utilis* enzyme at 80 ng/μl soluble pancreatin.

### The 7-Day Study

**Figure 1:**
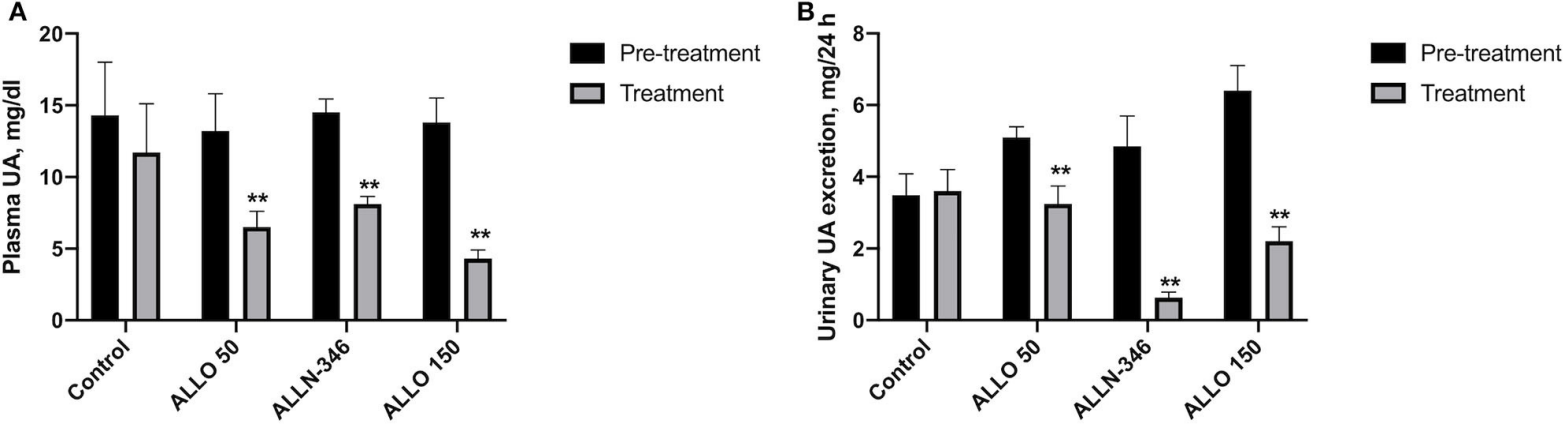
Plasma UA level **(A)** and urinary UA excretion **(B)** in uricase/UrOx deficient mice following the 7-day study. Data are shown as mean ± SEM. Control group, no treatment (*n* = 7); ALLO 50 group, allopurinol supplemented in drinking water (50 mg/L) (*n* = 8); ALLO 150 group, allopurinol supplemented in drinking water (150 mg/L) (*n* = 9); ALLN-346 group, ALLN-346 mixed with food provided *ad libitum* (*n* = 7). **Statistically significant differences considering *p* < 0.05 for comparison between pretreatment and treatment periods.

Removal of allopurinol from the drinking water in the 7-day study resulted in severe hyperuricemia and hyperuricosuria with mean values of plasma and urine urate of 14.01 ± 0.86 mg/dl and 5.03 ± 0.35 mg/24 h, respectively. Hyperuricemia was significantly reduced by 51% (from 13.20 ± 2.63 to 6.50 ± 1.10 mg/dl), 44% (from 14.50 ± 0.90 to 8.11 ± 0.53 mg/dl), and 69% (from 13.81 ± 1.73 to 4.31 ± 0.59 mg/dl) in the ALLO 50, ALLN-346, and ALLO 150 groups, respectively (*p* < 0.05). No changes in plasma UA concentrations were observed in the control group (Figure 1A).

Daily urate excretion was significantly reduced by 35% (from 5.01 ± 0.25 to 3.24 ± 0.50 mg/24 h), 87% (from 4.85 ± 0.85 to 0.63 ± 0.15 mg/24 h), and 66% (from 6.38 ± 0.65 to 2.20 ± 0.41 mg/24 h) from pretreatment in the ALLO 50, ALLN-346, and ALLO 150 groups, respectively (*p* < 0.05). No changes in daily urate excretion were observed in the control group (Figure 1B).

Pre-randomization data for food and water intake and daily urine volumes are provided in Table 1. Food intake during the treatment period was similar between the experimental groups; however, it was significantly higher when compared to the intake during the pretreatment period (*p* < 0.05). The mean water intake during the pretreatment period was 11.93 ± 0.75 ml/24 h (Table 1). Although water consumption remained stable in the control and ALLN-346 groups during treatment, a significant (*p* < 0.05) decrease in water intake was observed in the ALLO 50 and ALLO 150 groups (Table 1). The mean urine volume during the pretreatment period was 7.45 ± 1.01 ml/24 h (Table 1), and during treatment, an almost 2-fold decrease in daily urine volume was observed in the control, ALLO 50, and ALLO 150 groups (*p* < 0.05) although no changes in urine volume were observed in the ALLN-346 group.

**Table 1:** Pre- and post-treatment daily food and water intake as well as urine output in uricase/UrOx deficient mice for both the 7- and 19-day studies.

**Groups**	**Food intake (g)**		**Water intake (ml)**		**24 h urine volume (ml)**	
	**Pre-treatment**	**Treatment**	**Pre-treatment**	**Treatment**	**Pre-treatment**	**Treatment**
**7-day study**						
Control	2.54 ± 0.17^a^	3.20 ± 0.10^b^	11.93 ± 0.75^a^	10.60 ± 1.19^a, b^	7.45 ± 1.01^a^	4.30 ± 0.55^b^
ALLO 50		3.10 ± 0.07^b^		7.30 ± 0.66^b^		4.20 ± 0.53^b^
ALLO 150		3.20 ± 0.11^b^		7.90 ± 0.48^b^		4.10 ± 0.24^b^
ALLN-346		2.90 ± 0.08^b^		12.80 ± 0.73^a^		8.34 ± 0.98^a^
**19-day study**						
Control	3.80 ± 0.08	3.85 ± 0.16	14.59 ± 0.52^a^	17.26 ± 1.35^a^	7.87 ± 0.53^a^	11.12 ± 1.72^a, b^
ALLO 90		3.78 ± 0.18		14.73 ± 0.65^a^		8.17 ± 1.12^a, b^
ALLN-346		3.57 ± 0.13		18.49 ± 1.07^b^		12.75 ± 1.09^b^

*Data are shown as mean ± SEM. Control group, no treatment (n = 7); ALLO 50 group, allopurinol supplemented in drinking water (50 mg/L) (n = 8); ALLO 150 group, allopurinol supplemented in drinking water (150 mg/L) (n = 9); ALLN-346 group, ALLN-346 mixed with food provided ad libitum (n = 7). The different superscript letters describe statistically significant differences when p < 0.05, pre- vs. post-treatment*.

### The 19-Day Study

**Figure 2:**
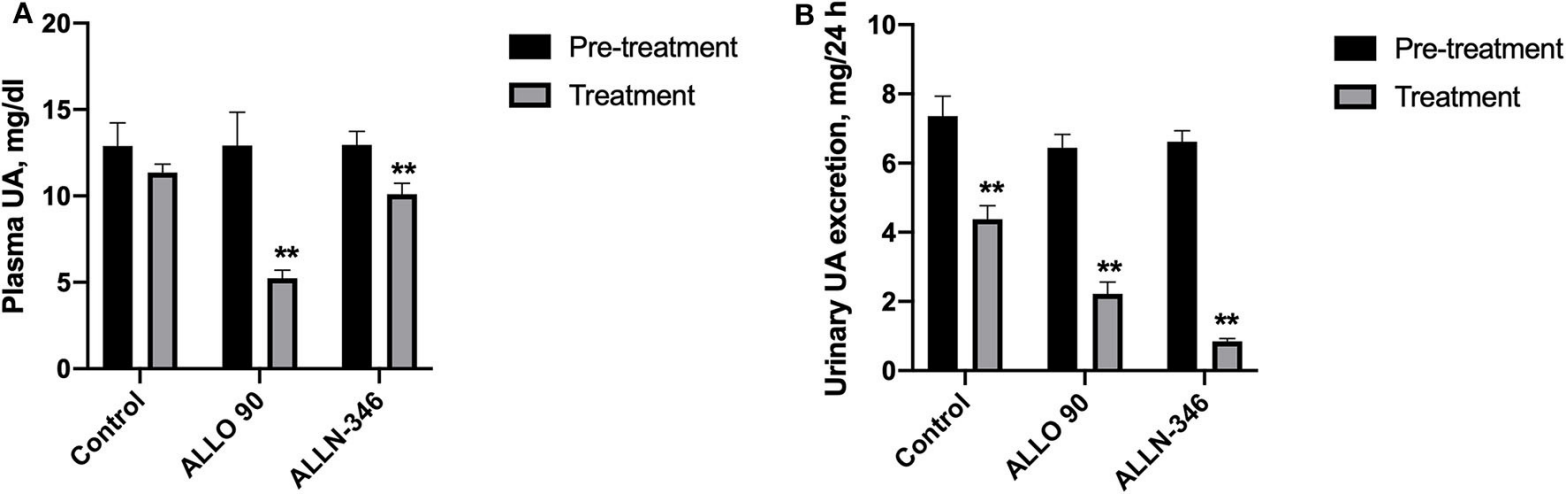
Plasma UA level **(A)** and urinary UA excretion **(B)** in uricase/UrOx deficient mice following the 19-day study. Data are shown as mean ± SEM. Control group, no treatment (*n* = 7); ALLO 90 group, allopurinol supplemented in drinking water (90 mg/L) (*n* = 7); ALLN-346 (*n* = 10), ALLN-346 mixed with food and provided for up to 6 h. **Statistically significant differences considering *p* < 0.05 for comparison between pretreatment and treatment periods.

Similar to the 7-day study, removal of allopurinol from the drinking water for a week prior to randomization resulted in the development of severe hyperuricemia and hyperuricosuria in mice with mean plasma and urine urate levels of 12.93 ± 0.72 mg/dl and 6.79 ± 0.24 mg/24 h, respectively. Plasma UA concentrations were significantly decreased (*p* < 0.05) by 62% (from 12.92 ± 1.92 to 4.93 ± 0.90 mg/dl) and 28% (from 12.96 ± 0.78 to 9.30 ± 0.70 mg/dl) in the ALLO 90 and ALLN-346 groups, respectively. No significant changes in plasma UA concentrations were observed in the control group (Figure 2A).

Urine urate excretion was significantly decreased (*p* < 0.05) by 72% (from 6.45 ± 0.38 to 1.82 ± 0.34 mg/24 h) and 88% (from 6.62 ± 0.31 to 0.82 ± 0.10 mg/24 h) in the ALLO 90 and ALLN-346 groups, respectively. Interestingly, a reduction in urine urate excretion of 46% (from 7.36 ± 0.58 to 3.97 ± 0.5 mg/24 h) was also observed in the control group (*p* < 0.05) (Figure 2B).

Declining renal function was observed during the 19-day treatment period with UA clearance significantly reduced in all experimental groups (*p* < 0.05) (Table 2), which is not unexpected in this mouse strain, which requires treatment with high doses of allopurinol for survival. Pretreatment plasma creatinine concentrations ranged between 0.30 and 0.34 mg/dl and, following randomization, significantly increased (*p* < 0.05) in all study groups (Table 2). This was reflected in the reduction of FEUA by 40 and 87% in the control and ALLN-346 groups, respectively (*p* < 0.05) during the 19-day treatment period (Table 2). No changes in FEUA were observed in the ALLO 90 group (Table 2).

**Table 2:** Pre- and post-treatment renal test parameters in uricase/UrOx deficient mice in the 19-day study.

**Group**	**Plasma creatinine (mg/dL)**		**UA clearance (mL/h)**		**Creatinine clearance (ml/h)**		**FEUA (%)**	
	**Pre-treatment**	**Treatment**	**Pre-treatment**	**Treatment**	**Pre-treatment**	**Treatment**	**Pre-treatment**	**Treatment**
Control	0.34 ± 0.05	0.43 ± 0.05^*^	2.58 ± 0.31	1.53 ± 0.17^*^	9.99 ± 2.06	10.32 ± 2.45	29.94 ± 4.91	18.0 ± 2.39^*^
ALLO 90	0.30 ± 0.06	0.49 ± 0.08^*^	2.43 ± 0.36	1.54 ± 0.24^*^	14.3 ± 3.27	11.57 ± 3.42	19.56 ± 2.24	19.90 ± 5.1
ALLN-346	0.32 ± 0.03	0.42 ± 0.03^*^	2.38 ± 0.24	0.39 ± 0.07^*^	10.16 ± 1.32	12.15 ± 1.70	26.54 ± 3.52	3.46 ± 0.58^*^

Data are shown as mean ± SEM. Control group, no treatment (n = 7); ALLO 90 group, allopurinol supplemented in drinking water (90 mg/L) (n = 7); ALLN-346 (n = 10), ALLN-346 mixed with food and provided for up to 6 h.

* *pDescribes statistically significant differences within groups when p < 0.05, pre- vs. post-treatment*.

**Figure 3:**
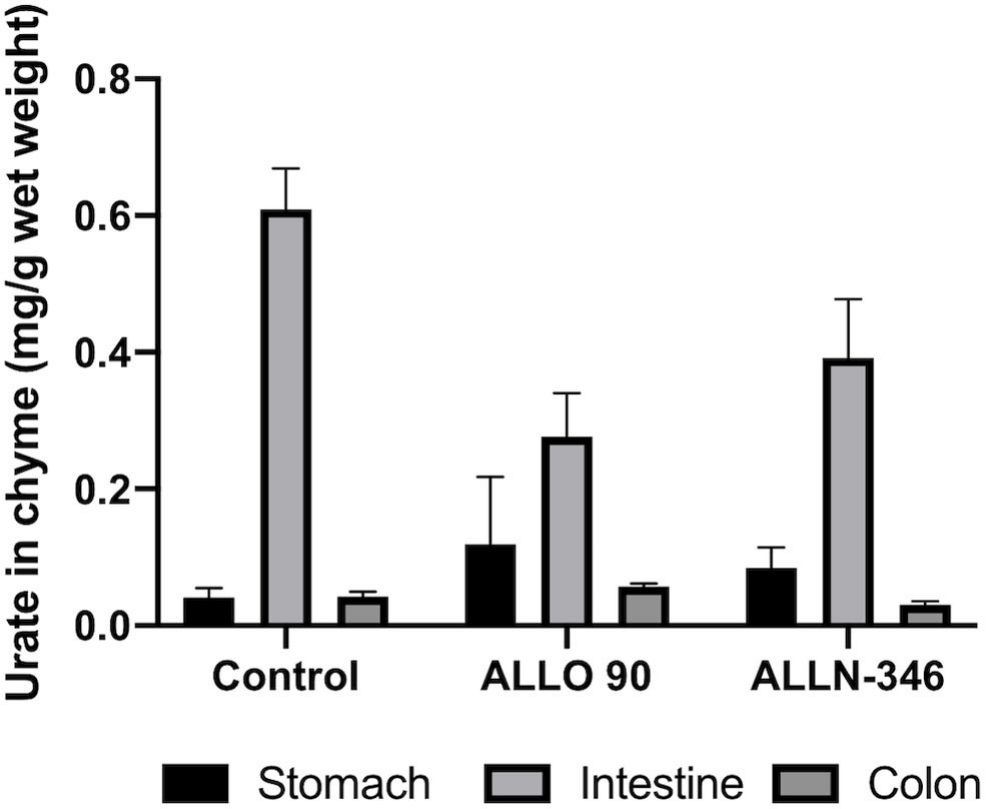
Urate levels in chyme samples from the gastrointestinal tract of uricase/UrOx deficient mice following the 19-day study. Data are shown as mean ± SEM. Control group, no treatment (*n* = 7); ALLO 90 group, allopurinol supplemented in drinking water (90 mg/L) (*n* = 7); ALLN-346 (*n* = 10), ALLN-346 mixed with food and provided for up to 6 h.

The mean range of urate in the chyme collected from the stomach and colon of the uricase/UrOx deficient mice was between 0.04 and 0.08 mg/g based on wet weight of digesta and was similar between groups. However, in the small intestine, the urate levels were several fold higher with the highest measured urate concentration observed in the control group (610 ± 0.060 mg/g wet weight), and lower levels of urate, by 29 and 55%, were recorded in the ALLN-346 and ALLO groups, respectively (Figure 3).

The food and water intake as well as daily urine volume are shown in Table 1. The food intake ranged between 3.6 and 4.0 g/day and was not significantly different between groups during both study periods. The water intake during the pretreatment period ranged between 13.5 and 16.0 mL/day and was not significantly different during the treatment period with the exception of the ALLN-346 group, in which it increased significantly by 27% (*p* < 0.05), compared to pretreatment values and that observed in the other study groups. Accordingly, a significantly increased daily urine production was observed in the ALLN-346 group following the 19-day treatment period compared to pretreatment values (*p* < 0.05) (Table 1).

## Discussion

The prevalence of hyperuricemia and gout has increased in recent years, and there is an unmet need for new therapies that help patients with refractory gout or CKD. We tested a potential new therapeutic option for patients with hyperuricemia and gout in URKO mice that mimics, to an extent, the disease in subjects with severe hyperuricemia and kidney impairment. The two current studies described were designed as seminal, proof-of-concept, preclinical investigations prior to initiating early clinical trials. Both the 7- and 19-day studies demonstrate that oral enzyme therapy with ALLN-346 resulted in a substantial reduction in the body urate burden as evident in the normalization of urate excretion and a significant reduction in plasma urate concentration. In the 7-day study, the mean reduction in plasma urate concentration was 44%, which was similar to the reduction achieved with the allopurinol dose of 50 mg/l. However, this reduction was higher in comparison to the mean reduction of 28% observed during the 19-day study. Because the daily ALLN-346 dose was similar between studies, ~2,000 units/day, the difference in the reduction of plasma urate observed was likely due to the dosing frequency of the ALLN-346. In the 7-day study, the ALLN-346 was provided with food *ad libitum*, throughout the entire day, although access to ALLN-346 in the 19-day study was restricted toa 4- to 6-h window at night, mimicking, to an extent, the effect of a single daily dose. Given our understanding of gut urate homeostasis (12, 29, 30), our findings suggest that secretion of UA in the intestine likely occurs over the entire day in the setting of severe hyperuricemia and impaired renal function.

Reduction in renal UA burden and excretion by ALLN-346 was a central finding in this seminal study. As demonstrated in both studies, UA excretion was normalized with the use of ALLN-346 or allopurinol. In these treatment groups, the normalization of UA excretion was rapid. Interestingly, the reduction in uric acid excretion in both the ALLO 50 and ALLO 150 groups was lower than that in the ALLN-346 group (88% in ALLN-346 group vs. 72% in ALLO group, *p* = 0.0198). We speculate that this difference is either a result of the dose of allopurinol or possible off-target effects of allopurinol. The major active allopurinol metabolite, oxypurinol, partially blocks the URAT1 transporter. By blocking URAT1, urate reabsorption from the renal proximal tubules is altered with a net result of increased urate excretion (11). In contrast, ALLN-346 is expected to degrade urate only within the intestine, including both the urate that is secreted into the intestine via extrarenal elimination and the urate formed in the intestine itself. Consequently, ALLN-346 is expected to affect the urate equilibrium across different compartments in the body (kidney, joints, blood, and intestine) and thereby reduce overall hyperuricemia. Thus, in contrast to other available ULTs, ALLN-346 does not directly affect the generation of urate within the body or function of different urate transporters in kidneys/intestine but rather degrades urate within the intestinal tract.

In the 19-day study, a significant increase in plasma creatinine was observed at the end of the study in all experimental groups. These results are not in agreement with previously published work by Lu et al. (31), in which after 2 weeks of allopurinol supplementation, at doses of either 40 mg/kg or 100 mg/kg, serum creatinine was reduced in comparison to the control, untreated group. Mean consumption of allopurinol in the current study was 57.4 mg/kg body weight and, thus, was within the range that was reported by Lu et al. (31). The differences observed between the two studies may be explained by differences in genetic background or age of the mice used or differences in study design with the underlying cause likely being multifactorial in nature.

The FEUA presents the ratio between UA and creatinine clearance and highlights the dependence of UA secretion on kidney function. It is well-known that, in patients with gout, FEUA is lower than that in healthy subjects (11, 32). Moreover, genetic mutations or polymorphisms in renal and intestinal urate transporters can alter FEUA (13, 33, 34). FEUA in healthy mice is normally within the range of 5% to 15% (23). This means that, in healthy animals, around 90% of urate that is filtered in the renal proximal tubules is reabsorbed back into circulation. The increased FEUA in URKO mice by ~30% is a consequence of severe hyperuricemia (5x that of normal) and hyperuricosuria (3x that of normal) due to the lack of hepatic uricase. Following ALLN-346 therapy in the 19-day study, the FEUA was normalized, driven by reductions in both urine and plasma UA concentrations. Interestingly, in the ALLO 90 group, no differences in FEUA were noted between pretreatment and treatment periods despite a reduction in both plasma and urine urate concentrations. Similar results were reported in a human clinical trial in which gout patients treated with allopurinol displayed unchanged FEUA despite a reduction in sUA concentration (11). This result is complicated and may be related to oxypurinol, which acts as a substrate for the URAT1 transporter and competes against urate reabsorption, thereby causing an additional increase in uric acid excretion that can result, as seen in our study, in a small increase or no change in FEUA. Increases in plasma creatinine concentrations were recorded in all groups at the end of the study. The highest plasma creatinine concentration was recorded in the ALLO 90 group. This increased plasma creatinine could offset creatinine clearance in a manner not seen in the other two groups.

To further confirm the mechanism of action of ALLN-346, the concentration of urate was analyzed in chyme samples obtained from various parts of the gastrointestinal tract of the uricase/UrOx deficient mice. A low amount of urate, which was similar between groups, was observed in the digesta obtained from the stomach and colon, which was in agreement with previously published results (35). However, urate levels were several fold higher in the digesta obtained from the small intestine, which is consistent with its role as the primary site of extrarenal urate elimination (23, 36). With both ALLN-346 and allopurinol therapies, chyme urate levels were reduced, in line with the reduced plasma urate levels observed; the higher the plasma urate, the higher the urate concentration in the chyme from the small intestine, supporting the notion that the small intestine plays an active role in urate elimination under conditions of severe hyperuricemia.

Unlike existing effective, injectable uricase therapy containing pegloticase, which can cause infusion reactions and anaphylaxis, ALLN-346 is designed specifically as an oral enzyme therapy that acts only within the intestinal lumen, and it is not expected to be absorbed. ALLN-346 is 137 kD in size and, thus, is too large for passive absorption into circulation and with no known mechanism involving receptor binding to facilitate active absorption. Thus, systemic absorption into circulation or possible immunogenicity is not expected. This was indirectly shown in both the 7- and 19-day studies, in which oral therapy with ALLN-346 had no adverse effects on mouse well-being and behavior. The mice maintained normal food and water intake as well as body weight, all of which were similar to that observed in the ALLO and control groups. ALLN-346 functions to degrade urate that is secreted from circulation (extrarenal elimination) or that is formed in the intestine itself. It is expected that, over time, oral therapy with ALLN-346 enhances enteric elimination of urate, preventing its reabsorption back into circulation, and thereby reducing serum urate concentrations and overall urate burden to the kidneys and other tissues/organs in patients that have hyperuricemia with gout and/or CKD. ALLN-346 catalyzes the conversion of urate to soluble allantoin, carbon dioxide, and hydrogen peroxide (H_2_O_2_). It is expected that soluble allantoin is excreted by the kidneys, and H_2_O_2_ is degraded by catalase present in the intestinal mucosa (37). Also, because ALLN-346 is a protein, it is expected that the majority of the enzyme is excreted via the stool or a portion is digested to peptides and eventually amino acids, similar to food proteins that are subsequently reutilized for protein synthesis in the body. Systemic parenteral and subcutaneous administration of recombinant uricases, even those that are PEGylated, profoundly lowers sUA. However, this approach is markedly limited by the immunogenicity of uricases, which are foreign proteins to humans (38). Uricase therapy, exemplified by pegloticase, frequently stimulates development of treatment-emergent antibodies that alter drug pharmacokinetics and, within weeks to months, limit the efficacy of uricase therapy. Whether ALLN-346 is immunogenic like pegloticase and rasburicase and whether potential immunogenicity of ALLN-346 and mucosal immunity processes could impact the long-term efficacy of ALLN-346 remain to be determined in future clinical studies.

Some of the limitations of our studies include that only a single dose of ALLN-346 was tested under variable circumstances with one study providing access to the drug over the entire 24 h cycle versus in the other, access to the ALLN-346 limited to up to 6 h at night. Moreover, an initial formulation that was not optimized for gastric survival was utilized in these studies. The described studies were designed as seminal, proof-of-concept, preclinical investigations prior to initiating early clinical trials. Because this concept is novel, it requires initial proof of biologically significant activity. Clearly, the current work demonstrates that machine learning–guided molecular evolution can enable the optimization of uricase stability and activity in the intestinal tract and, when administered as oral enzyme therapy, can reduce both plasma and urine uric acid solely through the degradation of urate that is secreted from circulation or formed in the gut itself. Moreover, there is room for the initial ALLN-346 formulation to be optimized further for human clinical trials to ensure maximal survival of the formulation in the acidic pH of the stomach. Possible enteric coating of the ALLN-346 in an oral dosage form could maximize the survival of the enzyme prior to it reaching the target site of the small intestine, where extrarenal elimination of urate from circulation is modulated by the ABCG2 and GLUT9 transporters.

The oral therapy with ALLN-346 was well-tolerated throughout the study as evidenced in the animals' normal food and water intake, growth, and behavior. The therapy resulted in significant reduction of plasma urate and normalization of urine uric acid excretion. The effect on plasma urate reduction was similar to that observed following ALLO administration at a dose of 50 mg/l, and the effect on urine uric acid excretion was superior to that observed following administration of the maintenance dose of ALLO at 150 mg/l, which is required to sustain this animal model. The underlying physiology of hyperuricemia and the extra-renal pathway of uric acid elimination corresponds to the ALLN-346 mechanism of action of the degradation of urate, specifically along the intestinal tract. Thus, ALLN-346 has the potential for use in single or combination drug ULT regimens for treatment of hyperuricemia in gout with and without compromised kidney function.

## Data Availability Statement

All datasets presented in this study are included in the article/supplementary material.

## Ethics Statement

The animal study was reviewed and approved by University of Lund Ethics Review Committee on Animal Experiments.

## Author Contributions

KP, AD, RT, ES, and DG were responsible for preparing the manuscript. KP, AD, NM, PS, and SP were involved in performing the study. KP, AD, NM, and PS conducted data analyses for the study. AD, RT, SP, and DG designed the study, interpreted the results, and critically reviewed the manuscript. All authors contributed to manuscript revision and have read and approved the submitted version.

## Conflict of Interest

RT has a research support from Astra-Zeneca and is consulting to Selecta, Sobi, and Horizon. KP is employed in SGP+Group, Sweden. AD and DG are employed in Allena Pharmaceuticals, USA and RT served as a paid consultant to Allena Pharmaceuticals, USA. SP is the owner of SGP+Group, Sweden. The remaining authors declare that the research was conducted in the absence of any commercial or financial relationships that could be construed as a potential conflict of interest. The authors declare that this study received funding from Allena Pharmaceuticals. The funder was involved in the study design, data analysis, writing of this article and the decision to submit it for publication.
